# Impaired plasma lipid profiles in acute hepatitis

**DOI:** 10.1186/1476-511X-9-5

**Published:** 2010-01-23

**Authors:** Libo Luo, Xiangke Pu, Yongzhong Wang, Ning Xu

**Affiliations:** 1 First People's Hospital of Changzhou, Jiangsu 213003, China; 2 Third People's Hospital of Changzhou, Jiangsu 213001, China; 3 School of Radiology and Public Health, Soochow University, Jiangsu 215123, China; 4 Department of Clinical Chemistry and Pharmacology, University Hospital of Lund, Lund S-22185, Sweden

## Abstract

The present study examined plasma lipid profiles in thirty patients suffered from acute viral hepatitis. Patients' blood samples were collected at both the debut and recovery of diseases. Thirty sex and age matched normal subjects were included as controls. Plasma total triglycerides (TG), total cholesterol, high density lipoprotein cholesterol (HDL-C), low density lipoprotein cholesterol (LDL-C), apolipoprotein AI (ApoAI), apolipoprotein B (ApoB), lipoprotein (a) (Lp(a)), blood coagulation status including prothrombin complex activity and activated partial tromboplastin time (APTT), and hepatic functions were determined by the automatic biochemical analytical instrument. It demonstrated that plasma levels of total cholesterol, HDL-C and apoAI were significantly lower in the patients at the acute phase of hepatitis than those in normal subjects, whereas plasma levels of TG and LDL-C were obviously higher in the patients than in normal subjects (P < 0.05). Moreover, we demonstrated that patients' plasma levels of total cholesterol, LDL-C, HDL-C and apoAI were lower at the active phase of the diseases than at the recovering phase, which indicating that acute liver damage could significant influence lipid metabolism in vivo. No pathological changes of blood coagulation status occurred in these patients during the study as all selected patients had moderate hepatitis. It may conclude that examinations of plasma lipid profile could be considered as a clinical index to reflect liver damage in the active phase of hepatitis.

## Introduction

Liver is the most important organ for the metabolism of lipids, lipoproteins and apolipoproteins. Under normal circumstances, most plasma endogenous lipids and lipoproteins are synthesized in the liver and then are secreted into the blood circulation [1,2]. And plasma lipoproteins are also mainly catabolism by the liver to maintain the relative balance of lipid and lipoprotein metabolism in vivo [3]. It has been well documented that chronic liver dysfunction might interfere lipid metabolism in vivo and could change plasma lipid and lipoprotein patterns [4]. Acute hepatitis may be referred to an inflammatory process of the liver lasting less than six months. In China, the most common etiology of acute hepatitis is viral infection, in which hepatitis A and hepatitis E are the most common causes. In clinical, the courses of acute hepatitis may vary widely from mild symptom that does not require treatment to the fulminant hepatic failure that needs emergency liver transplantation. Acute viral hepatitis is more likely to be asymptomatic in younger people. In addition, acute hepatitis may occur less commonly with infections such as Epstein-Barr virus, cytomegalovirus, adenovirus, herpes simplex and Coxsackie virus or with other noninfectious reasons. It has been demonstrated that in the acute and/or chronic liver diseases, hepatic function could be impaired and the circulating lipids and lipoproteins are not only present in abnormal amounts but they frequently also have abnormal composition including electrophoretic mobility and appearance [4]. Previous studies pay more emphasis on changes of lipid metabolisms under chronic hepatitis and cirrhosis with or without hepatocellular carcinoma [5-7]. In the present study we followed plasma lipid and lipoprotein patterns of patients suffered from acute hepatitis to further explore the changes of lipid and lipoprotein profiles of the patients.

## Materials and methods

### Patients and normal subjects

Thirty cases of acute hepatitis patients who were admitted to the hospital's inpatients were subjected in the present study, 21 males and 9 females, aged from 19 to 81 years old. All patients had moderate hepatitis. Clinical diagnosis was applied according to the diagnostic criteria of "Viral Hepatitis Prevention Program" of the Chinese Society of Hepatology and Society of Infectious Diseases [8]. The characterization of the patients is listed in the table 1. Data concerning virus antigen and antibody, and viral loads in blood is shown in the Table 2. And there was no any patient with HIV positive in the present study. There was no patient received anti-viral drug therapy, blood transfusions or plasmapheresis. Duration of hospitalization all patients were given liver protective therapy and advised to have proper rest and go on rational diet. Some patients were given traditional Chinese medicine to improve liver function. In the present study there was no patient underwent liver biopsy. And up to now there was no patient was found to develop chronic active hepatitis. Thirty healthy subjects, 16 males and 14 females, aged from 20 to 63 years old, were included as controls. All normal subjects were confirmed by the negative results of serum biochemical tests, virus tests and B-type ultrasonic inspection to exclude hepatitis or other chronic liver diseases and metabolic diseases. Fasting blood samples were obtained and preserved at -70°C before further examination. The present study was approved by the local Ethics Committee.

**Table 1:** Characterization of patients and controls

	Patients	Control
**Cases**	30	30
**Ages (Means ± SD)**	41.7 ± 17.3	46.0 ± 13.6
**Sex (male/female)**	21/9	16/14
**Hospitalization (days)**	24.6 ± 15.3	Medical normal
**ALT (IU/L)**	1227.9 ± 690.7	
**Clinical diagnosis**	25 cases of acute jaundice viral hepatitis, 5 cases of non-jaundice	
**Discharged**	Clinical cure	
**Infection**	19 cases of HEV, 4 cases of HBV and seven cases of non-A non-E	
**Ultrasound examines**	10 cases of hepatomegaly, 4 cases of hepatic cyst and 1 case hepatic hemangioma	

**Table 2:** Viral tests of the patients

Virus	Positive cases	Viral load (copies/ml)	HBsAg	Antibody against virus
**HBV**	4	3.61 × 10^6 ^± 6.30 × 10^6^	+	-
**HEV**	19	unknown	-	+
**non-A non-E**	7	-	-	-
**HIV**	0	-	-	-

### Laboratory analyses

Blood was collected from all subjects under standardised conditions after overnight fasting. Triglycerides (TG) and total cholesterol were determined by the endpoint detection (GPO-PAP Method, CHO-PAP Method). ApoA, apoB as well as Lp(a) were determined by the immunological turbidimetric method. All laboratory parameters were directly determined on an automatic biochemical analyzer (Olympus AU2700, Japan). Patients' coagulation status including prothrombin complex activity and activated partial tromboplastin time (APTT), were determined as a routine test and patients' coagulation status was at normal ranges.

### Statistical analysis

Statistical analysis was performed with the GraphPad Prism version 5. Data are expressed as means ± SD. Differences between patients and healthy subjects were compared by the non-paired t-test. Lipid profiles in patients under different diseases phases were analyzed by the one-way ANOVA followed by the paired t-test. A p value less than 0.05 (P < 0.05) was considered as significant.

## Results

**Figure 1:**
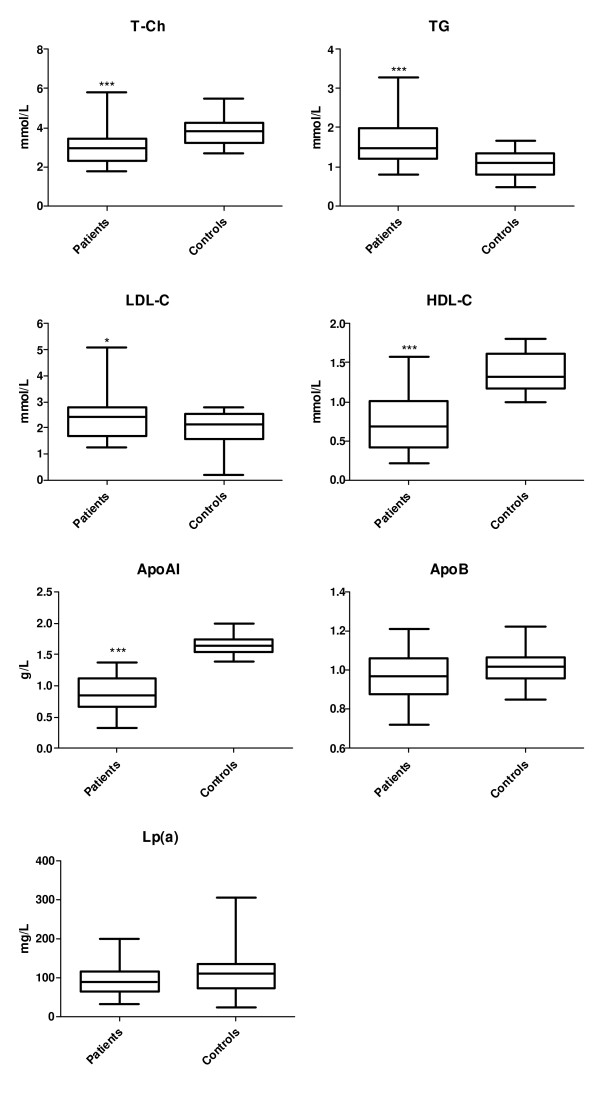
**Plasma lipid levels in acute hepatitis patients and in normal subjects**. Data are mean ± SD. * P < 0.05 and *** P < 0.001 vs. normal subjects.

**Figure 2:**
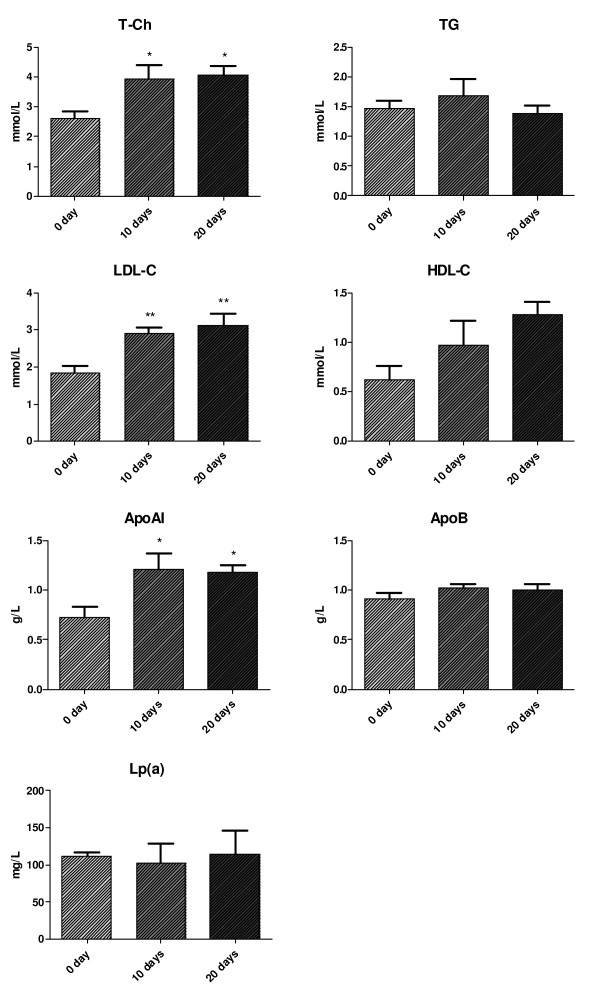
**Lipid profiles in patients at different phases of the diseases**. Day 0 represents the debut of disease, day 10 and day 20 represent the treatment days. Data are mean ± SD. * P < 0.05 and ** P < 0.01.

As shown in figure 1, serum levels of total cholesterol, HDL-C and apoAI were significantly lower in the acute hepatitis patients compared to the normal subjects, whereas serum levels of TG and LDL-C were obviously higher in the patients than in normal subjects (P < 0.05). Moreover, we demonstrated that patients' serum levels of total cholesterol, LDL-cholesterol, HDL-cholesterol and apoAI were lower at the active phase of the diseases than at the recovering phase (Figure 2). There is no statistical significant correlation between the plasma viraemia and the changes of lipid profiles (data not shown).

## Discussion

Liver is one of the most important organs for the metabolism of plasma apolipoproteins, endogenous lipids and lipoproteins. Most plasma apolipoproteins, endogenous lipids and lipoproteins are synthesized by the liver [1,2], which depends on the integrity of cellular functions of liver [2,3]. As in blood circulation lipids do not dissolve in plasma, they need in combination with different apolipoproteins to form lipoproteins that may transfer endogenous or exogenous lipids to different organs or tissues for further metabolism. Under normal physiological conditions, liver plays an important role to regulate lipid and lipoprotein metabolisms. Liver not only synthesizes and secretes endogenous lipoprotein, synthesis of key enzyme for the LDL metabolism, i.e., lecithin cholesterol acyltransferase (LCAT), hepatic lipase and apolipoproteins, but also regulates catabolism of various plasma lipoproteins via hepatic cellular surface lipoprotein receptors, which may maintain relative equilibrium of plasma lipids and lipoproteins in vivo [9,10]. These processes could be interfered or impaired when hepatic cellular damage, which leads to an alteration of plasma lipid and lipoprotein patterns. And syntheses of cholesterol, triglycerides, apoAI, apoB and Lp(a) could be changed and their plasma concentrations will be altered correspondingly [11]. Therefore, examination of plasma lipid and lipoprotein levels will be helpful to evaluate the extent of the hepatic damage. It is well known that plasma levels of cytokines, lipid peroxides and anti-oxidant status could be changed under acute or chronic hepatitis [12-14], which may also interfere the lipid metabolism in vivo.

In the present results we demonstrated that plasma levels of total cholesterol, HDL-C and apoAI were significantly lower in the patients at the acute phase of hepatitis than those in normal subjects, whereas plasma levels of triglycerides and LDL-C were obviously higher in the patients than in normal subjects. And patients' plasma levels of total cholesterol, LDL-C, HDL-C and apoAI were lower at the active phase of the diseases than at the recovering phase, which indicating that acute liver damage could significant influence lipid metabolism in vivo. It is well known that severe acute hepatitis is an serious acute inflammatory process that leads to a hepatic cellular necrosis [15]. It has been reported when acute hepatitis occurred, liver function could be severely damaged, which could significantly inhibit hepatic apoAI synthesis [16]. Serum levels of apoAI, apoAII and total cholesterol were negatively correlated to serious of liver damage [17-19]. According to our results and together with the literatures [20,21] apoAI and HDL-C could be considered as the best indicator to evaluate accurately the liver damage. The combination of plasma levels of apoAI and HDL-C together with prothrombin activity and serum levels of bilirubin and transaminase could be a best index for evaluating the prognosis of acute hepatitis.

## Competing interests

The authors declare that they have no competing interests.

## Authors' contributions

Conceived and designed the experiments: NX and LL. Performed the experiments: XP and YW. Analyzed the data: LL, XP, YW and NX. Wrote the paper: XP and NX. All authors read and approved the final manuscript.

## References

[B1] Bell AW Lipid metabolism in liver and selected tissues and in the whole body of ruminant animals Progress in lipid research 1979 18 3 117 164 10.1016/0163-7827(79)90013-4 396532

[B2] Tietge UJ Boker KH Bahr MJ Weinberg S Pichlmayr R Schmidt HH Manns MP Lipid parameters predicting liver function in patients with cirrhosis and after liver transplantation Hepato-gastroenterology 1998 45 24 2255 2260 9951906

[B3] Sherlock S Alcoholic liver disease Lancet 1995 345 8944 227 229 10.1016/S0140-6736(95)90226-0 7823717

[B4] Miller JP Dyslipoproteinaemia of liver disease Baillieres Clin Endocrinol Metab 1990 4 4 807 832 10.1016/S0950-351X(05)80080-1 2082907

[B5] Jiang J Nilsson-Ehle P Xu N Influence of liver cancer on lipid and lipoprotein metabolism Lipids in health and disease 2006 5 4 10.1186/1476-511X-5-4 16515689 PMC1420303

[B6] Katsuramaki T Mizuguchi T Kawamoto M Yamaguchi K Meguro M Nagayama M Nobuoka T Kimura Y Furuhata T Hirata K Assessment of nutritional status and prediction of postoperative liver function from serum apolioprotein A-1 levels with hepatectomy World journal of surgery 2006 30 10 1886 1891 10.1007/s00268-005-0590-z 16983478

[B7] Gupte P Dudhade A Desai HG Acquired apolipoprotein B deficiency with chronic hepatitis C virus infection Indian J Gastroenterol 2006 25 6 311 312 17264435

[B8] Chinese society of hepatology and society of infectious diseases, CMA. Hepatitis prevention programs China liver disease magazines 2000 8 3 324

[B9] Pangburn SH Newton RS Chang CM Weinstein DB Steinberg D Receptor-mediated catabolism of homologous low density lipoproteins in cultured pig hepatocytes The Journal of biological chemistry 1981 256 7 3340 3347 6259162

[B10] Dessi S Batetta B Pulisci D Spano O Anchisi C Tessitore L Costelli P Baccino FM Aroasio E Pani P Cholesterol content in tumor tissues is inversely associated with high-density lipoprotein cholesterol in serum in patients with gastrointestinal cancer Cancer 1994 73 2 253 258 10.1002/1097-0142(19940115)73:2<253::AID-CNCR2820730204>3.0.CO;2-F 8293385

[B11] Booth S Clifton PM Nestel PJ Lack of effect of acute alcohol ingestion on plasma lipids Clinical chemistry 1991 37 9 1649 1893600

[B12] Nanji AA Jokelainen K Rahemtulla A Miao L Fogt F Matsumoto H Tahan SR Su GL Activation of nuclear factor kappa B and cytokine imbalance in experimental alcoholic liver disease in the rat Hepatology 1999 30 4 934 943 10.1002/hep.510300402 10498645

[B13] Peterhans E Reactive oxygen species and nitric oxide in viral diseases Biol Trace Elem Res 1997 56 1 107 116 10.1007/BF02778986 9152514

[B14] Wang CC Cheng PY Peng YJ Wu ES Wei HP Yen MH Naltrexone protects against lipopolysaccharide/D-galactosamine-induced hepatitis in mice J Pharmacol Sci 2008 108 3 239 247 10.1254/jphs.08096FP 19023176

[B15] Vergani C Trovato G Delu A Pietrogrande M Dioguardi N Serum total lipids, lipoprotein cholesterol, and apolipoprotein A in acute viral hepatitis and chronic liver disease Journal of clinical pathology 1978 31 8 772 778 10.1136/jcp.31.8.772 690242 PMC1145405

[B16] Fukushima N Yamamoto K Ozaki I Sakai T [Apolipoprotein A-I, E, C-III and LDL-receptor mRNA expression in liver diseases] Nippon rinsho 1993 51 2 407 413 8464155

[B17] Cordova C Musca A Violi F Alessandri C Iuliano L Apolipoproteins A-I, A-II and B in chronic active hepatitis and in liver cirrhotic patients Clinica chimica acta; international journal of clinical chemistry 1984 137 1 61 66 10.1016/0009-8981(84)90312-7 6421513

[B18] Vergani C Trovato G Pietrogrande M Crocchiolo P Dioguardi N [Behavior of total lipids, cholesterol, lipoproteins and apolipoprotein A in the blood of subjects with acute hepatitis and chronic hepatopathy] Minerva medica 1978 69 31 2081 2094 208025

[B19] Selimoglu MA Aydogdu S Yagci RV Low plasma apolipoprotein A-I level: new prognostic criterion in childhood cirrhosis? The Turkish journal of pediatrics 2001 43 4 307 311 11765160

[B20] Jorge AD Ponce G Milutin C Sanchez D Diaz M Perez R Esley C [Importance of apolipoproteins A-1 and B in acute viral hepatitis and hepatic cirrhosis] Acta gastroenterologica Latinoamericana 1986 16 1 39 46 3577619

[B21] Geiss HC Ritter MM Richter WO Schwandt P Zachoval R Low lipoprotein (a) levels during acute viral hepatitis Hepatology (Baltimore, Md 1996 24 6 1334 1337 10.1002/hep.510240602 8938156

